# Biography of a scientist with strength, substance, sincerity and service: Late N. Srinivasan (1962-2021)

**DOI:** 10.6026/97320630018600

**Published:** 2022-06-30

**Authors:** R Sowdhamini

**Affiliations:** 1 National Centre for Biological Sciences (NCBS-TIFR), GKVK Campus, Bangalore, Karnataka 560065, India; 2 Molecular Biophysics Unit, Indian Institute of Science, Bangalore 560012, India; 3 Institute of Bioinformatics and Applied Biotechnology, Bangalore 560100, India

**Keywords:** Science, scientist, Srinivasan, proteins, structure, analysis, function

## Abstract

Late N. Srinivasan belongs to the GN Ramachandran lineage of protein structural analysts. His role in the advancement of the structure based understanding of signal transduction, protein kinase analyses and host-pathogen interactions both developing and using Bioinformatics tools for protein-protein interactions, protein dynamics, remote homology detection and polypeptide stereochemistry is well documented in the literature. Thus, his contribution to the understanding of protein function through structural analysis, using computational models and tools, is exceptional.

## Schooling and College during 1979-1984:

N. Srinivasan (NS) was born on June 12, 1962 at Madras (now referred as Chennai), Tamilnadu, India. His high schooling was at Rajah Muthiah Higher Secondary School (in Raja Annamalai Puram, Chennai) during 1976 and 1979. He was interested in physics and excelled in Mathematics at school. NS joined the Jain College at Chennai and completed his undergraduate degree in General Physics (1979-1982). During this time he taught Mathematics to students in the neighbourhood with passion as a service to the society. NS was a natural in solving difficult riddles and Mathematics puzzles. However, playing cricket was his favourite game during his leisure time. He then completed his graduate degree in Biophysics at the Madras University (1982-84). His interaction with popular Biophysics scientist like R. Srinivasan, N. Gautam and Vasantha Pattabhi during this period was an inspiration. Discussion on GN Ramachandran and his contribution to Biophysics dominated the discussion.

## Towards a doctorate degree during 1984-1991:

NS had applied for PhD both at Madras University and at Molecular Biophysics Unit (MBU), IISc but joined IISc in the lab of C Ramakrishnan (CR). CR was none other than the student of GNR, about whom NS had heard a lot during his graduate studies. This firmly established a link between NS and the GNR lineage of studying polypeptide stereochemistry. He also acquired excellent friends in MBU, who had both good academic minds and strength of character. With CR, he studied the backbone conformation of glycyl residues [1]. CR and NS had frequent conversations on many topics like computer programming, debugging, book-keeping and cricket! During his PhD, NS also collaborated extensively with P Balaram (PB). PB's collaborative intentions come with a strong purpose, namely to feed knowledge derived from the analysis of protein structures towards peptide design. But, NS could see the huge opportunity to think fluently and become a better researcher through collaborations. He was enthusiastic about discussions with PB. During discussions, NS often carried a notebook to note research ideas and came out beaming with happiness! It was during these collaborations that he met the author, Sowdhamini (Mini), who was a student of PB and later became NS's wife! His collaborations meant he broadened his research interests to study disulphide bond stereochemistry [2] and super secondary structures [3]. By this time, NS had grown into a mature scientist, with smart scientific thinking and known in MBU as a highly helpful and congenial colleague. He was also quite witty and enjoyed the company of his friends.

## Postdoctoral scholar during 1991-1998:

NS's postdoctoral tenure in Tom Blundell's (TLB) laboratory in the United Kingdom was for an equal seven years. TLB is a super-impressive personality and remained as someone NS admired until NS's very last days. He was funded by Tripos Associates initially (1991-94) and learnt about comparative modelling strategies [4-5]. NS also interacted with colleagues who worked on wet-lab biology and signal transduction. Indeed, some of his papers on pentraxin [6], PH domains [7], kinases [8-9] and protein symmetry [10] are noteworthy. He also learnt the collaborative skills by discussions with other labs in Birkbeck College, as opportunities were provided generously by TLB [11]. NS was amazingly productive and displayed original thinking. It was then he took the decision to step out and learn wet-lab biology in Mike Waterfield's laboratory (1994-1996). TLB and this lab had strong collaborations on signal transduction projects. He would often come in the evenings and continue his pursuits in structural bioinformatics. By 1996, TLB and few of his lab members moved from Birkbeck College to Cambridge, since TLB accepted the Chair position at the Biochemistry Department there. Whilst in Cambridge, NS rejoined TLB's laboratory and served there until 1998. TLB was pleased with NS’s productivity but was equally supportive of his seek for independence. His well-wishers were thrilled when NS was offered Assistant Professor Position in the same Department where he earned PhD, namely MBU, IISc. His wife was also lucky enough to find a faculty position in National Centre for Biological Sciences in the same city. The time spent in the UK for the couple was memorable, due to good research and the couple were also blessed with their daughter Jayashree.

## Starting as a scientist at IISc, India during 1998-2008:

With an independent position in MBU in 1998, NS did not waste any time and started putting his creative mind into practise. The initial contributions from his lab on Pairwise Alignment Database [12], genome-wide survey of human tyrosine Kinases [13- 15] and analysis of domain architectures [16] are examples of this kind. He had also collaborated with several biologists within the Institute [17-18] and in the country [19-20]. He received his National BioScience Award by the Department of Biotechnology, India in 2005 and also became a Fellow of Indian Academy of Sciences, Bangalore and National Academy of Sciences, Allahabad in 2007. Further, he received timely support in 1999 by obtaining Senior Research Fellowship by the Wellcome Trust, UK.

In 2004, his association with Bernard Offmann and Frederic Cadet of Universite de la Re Union of Re Union Islands began leading to their long-term collaboration in structural bioinformatics. He spent many summers as a Visiting Professor there with his family and they were looked after well every time. NS and Offmann published around 16 papers together on various themes such as protein structural variations and dynamics (for example [21]). NS's collaboration was also initiated with Alexandre de Brevern of INSERM, Paris and was supported by their Indo-French CEFIPRA grants, giving rise to 16 publications (for example, [22-23]). Together, NS co-mentored few students with both laboratories and these two lines of friendship flourished until the very end for NS. The year 2008 was memorable, since NS was conferred the Bhatnagar Award, the highest science prize in India. The Computational Biology community in India was pleased at this recognition and NS was the first in this area to be given this honour.

## Protein research during 2009-2018:

NS went on to apply his mind on several exciting projects. For instance, he worried about protein families that are fairly isolated and distant in sequence space [24]. He conceived ideas to design artificial sequence to bridge distantly related protein families, in collaboration with Sowdhamini's group. One of the beneficial effects that emerged was the realisation of new connections, where several domains of unknown function could be assigned reliable biological functions [25]. His interests also started moving into protein-protein interactions, fuelled by joint grants funded by UKIERI (including Jim Warwicker of Manchester UK and Pinak Chakrabarti of Bose Institute, India) and Centre of Excellence funded by Department of Biotechnology, India (with Nagasuma Chandra of IISc and Sowdhamini). There were several surprising findings such as the structural effects of binding in parts away from protein-protein interface [26] and the interface residues harbouring both intra- and inter protein interactions [27]. NS's group started focussing on large protein assemblies [28-29] and on host-pathogen interactions as well [30]. NS became a Fellow of National Academy of Science India, Allahabad in the year 2009. He was also one of the advisors for the Indian Bioinformatics Society. The years 2007-2011 were marked in NS’s career due to his memorable visits abroad (like China, Japan, Copenhagen, USA and Australia) and within India (like Kollam, Chandigarh, Jaipur, Trivandrum and Karaikudi). He would visit Institutes like University of Poona, Vellore Institute of Technology and few Indian Institutes of Technology. He had guided several PhD students in IISc and he was not slowing down.

## Revisiting the Ramachandran plot during 2018-2021:

NS could be often seen passionately explaining his re-visit of Ramachandran map, the work where he had collaborated with CR and one of his own students. Together, they showed that had we been conscious of small deviations from ideal peptide internal parameters of peptide bond, few regions of the Ramachandran map will move from being disallowed to allowed regions [31]. Many things excited NS about this project. The whole idea he had conceptualised, the combination of people involved (late GNR, late CR, himself and Ashraya his student thereby) four generations of scientists in the GNR school! Besides, nearly 2000 Ramachandran map calculations, accounting for deviations in bond lengths and angles of the peptide bond, were performed this time on computers where late CR had written all the codes himself. NS's joy knew no bounds when one of the key manuscripts was accepted on Teachers Day!! Such was the respect he had for his PhD mentors. No one can forget the enthusiasm with which he ended his IISc Institute talk in February 2020 with this work.

During the last two years of NS, we both had been travelling to countries like Finland, Strasbourg, Malaysia, New Zealand, Australia and to cities within the country like IIT-Madras and Coimbatore (just before the lock-down). NS’s group, whilst analysing host-pathogen interactions did examine the possibility of previously approved drugs to be repurposed to address ailments such as Tuberculosis [32] and COVID-19 [33]. Alas, he fell sick in December 2020 due to heart ailment and his health went through a decline. However, he published 19 papers since that time until his end. Whilst in the hospital, it was announced that he is conferred the Rustum-Chowski award for teaching and elected as fellow of the Indian National Science Academy, India. Despite his failing health, with less mobility at home or connections in the hospital, he continued to try his best to move academic matters. He had thoughtfully encouraged half of his lab members to apply for postdoctoral and higher positions, without giving them the doubt or fear that he may not survive.

## Exceptional qualities of Late N. Srinivasan:

NS never believed in strict discipline to achieve academic excellence. Instead, he taught people to enjoy whatever they were doing or learning. The coordination of our large conference, Asia Pacific Bioinformatics Conference in 2010 by NS, with nearly 600 participants and more than 25 volunteers, was an excellent example of NS's leadership qualities and organisational skills. NS develops regard for people when he finds them doing their work sincerely and with excellence. It does not matter what type of profession they are in. On the contrary, his blood boils if someone took their work lightly or did a half-hearted job. He himself takes every work assigned with great attention and meticulous mind. He gives excellent seminars and conveys protein structural principles with great clarity in his classes. He has taken extraordinary steps of working behind the scene in several instances, keeping the career and well-being of colleagues and lab members in mind. He is a strong supporter of women in science and had constantly been motivating his wife to do well. He is generous and fair-minded in providing due compliments to people and can motivate them to the right path with a single conversation. He often speaks from his heart and knows to treat every person special. He has been a pillar of selfless support to several distressed individuals. He can see people in distress easily and would go out of his way to help them – be it simply listening or giving them sound advice or words of encouragement. All through his ailment in the hospital, he displayed courage, found solace in hearing good music and fought really hard until his end. Trust we can take his legacy forward and his soul will rest in peace. Indeed, the greatest tribute to our beloved Srinivasan is to carry on his spirit of positive vibrations and dedication to Bioinformatics.
